# Manner implicatures in large language models

**DOI:** 10.1038/s41598-024-80571-3

**Published:** 2024-11-24

**Authors:** Yan Cong

**Affiliations:** 1 School of Languages and Cultures, Purdue University, West Lafayette, 47907 USA; 2 Center on Aging and the Life Course, Purdue University, West Lafayette, 47907 USA

**Keywords:** Explainability, Large language models, Pragmatic reasoning, Semantics, Natural language understanding, Conversational implicatures, Computational science, Scientific data

## Abstract

In human speakers’ daily conversations, what we do not say matters. We not only compute the literal semantics but also go beyond and draw inferences from what we could have said but chose not to. How well is this pragmatic reasoning process represented in pre-trained large language models (LLM)? In this study, we attempt to address this question through the lens of manner implicature, a pragmatic inference triggered by a violation of the Grice manner maxim. Manner implicature is a central member of the class of context-sensitive phenomena. The current work investigates to what extent pre-trained LLMs are able to identify and tease apart different shades of meaning in manner implicature. We constructed three metrics to explain LLMs’ behavior, including LLMs-surprisals, embedding vectors’ similarities, and natural language prompting. Results showed no striking evidence that LLMs have explainable representations of meaning. First, the LLMs-surprisal findings suggest that some LLMs showed above chance accuracy in capturing different dimensions of meaning, and they were able to differentiate neutral relations from entailment or implications, but they did not show consistent and robust sensitivities to more nuanced comparisons, such as entailment versus implications and equivalence versus entailment. Second, the similarity findings suggest that the perceived advantage of contextual over static embeddings was minimal, and contextual LLMs did not notably outperform static GloVe embeddings. LLMs and GloVe showed no significant difference, though distinctions between entailment and implication were slightly more observable in LLMs. Third, the prompting findings suggest no further supportive evidence indicating LLM’s competence in fully representing different shades of meaning. Overall, our study suggests that current dominant pre-training paradigms do not seem to lead to significant competence in manner implicature within our models. Our investigation sheds light on the design of datasets and benchmark metrics driven by formal and distributional linguistic theories.

## Introduction

The rise of large language models (LLMs) has led to significant advancements in natural language processing (NLP), enabling models to perform a wide range of tasks with impressive accuracy. However, despite these strides, their ability to handle pragmatic reasoning, such as manner implicatures, remains relatively under-explored. Manner implicatures occur when the use of an utterance conveys pragmatically enriched meaning beyond its literal semantics, as in cases where using “I was not unaware” implies more than its unmarked semantically equivalent counterpart “I was aware”^1–3^. Previous research has focused primarily on scalar implicatures, leaving manner implicatures, triggered by features such as negation, modality, and causality, less studied^4–7^. This study attempts to address that gap through explaining and evaluating pragmatic competence in Large Language Models (LLMs), in particular, LLMs’ representation and interpretation of manner implicatures. Specifically, the current work organizes metrics by the different levels at which they operate in a model: probabilities, embeddings, and generated text. We derived surprisal metrics based on negative log-probabilities, measured (cosine) similarities of embeddings, and used natural language prompts to evaluate LLM outputs. A series of experiments involving these metrics were conducted, targeting manner implicatures triggered by causal constructions, modal verbs, and negation.

First, the surprisal scores generated by various LLMs served as proxies for LLMs’ efficacy to recognize manner implicatures. Our results indicate that most LLMs performed at or below chance levels in detecting these implicatures. These findings suggest that while LLMs may be adept at handling surface-level linguistic patterns, their capacity to interpret more sophisticated, context-dependent pragmatic cues remains limited^5,7,8^. The results are particularly concerning in cases where understanding such subtleties is crucial, such as in human-computer interaction or nuanced language translation tasks.

Further, driven by the observations and proposals in formal semantics and pragmatics, we hypothesize that (cosine) similarity in distributional semantics reflects the degree of overlap in the meaning conveyed by different sentence pairs. Specifically, we propose that entailment tends to exhibit higher similarity, because the meanings of the sentences are closely aligned, with one sentence often including or reinforcing the information of the other. Further, we propose that implicatures may have lower similarity compared to entailments, as the connection between the sentences is more context-dependent and less directly tied to shared word meanings. Additionally, neutral sentence pairs would hypothetically have very low similarity, as there is little to no overlap in their semantic content. And equivalent sentence pairs are predicted to have very high similarity, since the two sentences are logically equivalent. We tested this hypothesis on a range of encoder-, decoder-, encoder-decoder transformer LLMs, and our findings suggested that not all LLMs showed similarity patterns that align with formal linguistics predictions. LLMs did not always generate patterns that are interpretable under formal semantics pragmatics theories.

We additionally explained and evaluated LLMs’ behavior using prompting. Findings suggested that more recent LLMs did not show significant improvement on representing manner implicatures. We speculate that the models’ overall performance could be attributed to several factors, including a lack of targeted training data for implicatures and limitations in their pretraining tasks and architectural design. To improve their ability to process manner implicatures, future LLMs must incorporate more sophisticated semantic and pragmatic reasoning frameworks and rely on carefully curated datasets that emphasize subtle context-sensitive inferences^2,3^.

## Background

### Assessment of LLMs’ pragmatic competence

Assessment of LLMs spans multiple domains, ranging from law, healthcare, to education, and so on^9^. However, we have relatively limited knowledge of what pragmatic capacity LLMs have. Only a few studies in NLP focused on pragmatic phenomena such as conversational implicatures^5–7^, and there are relatively few benchmarks on manner implicatures. One of the first works was IMPPRES^4^, a dataset of 25,000 sentence pairs annotated with presuppositions and scalar implicatures. Other studies were dedicated to the intersection of pragmatics and other phenomena, such as discourse connectives^8^, scalar implicatures and human cognition^10,11^, conjunction buttressing and natural language inference^12^, pragmatic benchmark in multilingual settings^13–16^.

The closest to the current work is about a subtype of manner implicature called “evaluativity implicature”^7^. When a human speaker says “Maria is tall”, they typically imply that Maria is considered tall from their perspective: *evaluated* by the speaker as tall. However, using a different construction, such as “Maria is taller than Sophie” does not necessarily mean that Maria is evaluatively tall; it could be the case that Maria is taller than Sophie in a context where both are still considered short. This raises the following questions: Can pre-trained LLMs “understand” evaluativity inference? How well can they distinguish the evaluative significance of different constructions in conversation? Additionally, would equipping LLMs with personas, such as being relaxed, sociable, or pragmatically skilled, improve their ability to handle implicit meaning? Cong^7^ offers a method for examining LLMs’ interpretation of evaluativity inferences by drawing on insights from experimental pragmatics and sociolinguistics. Cong^7^ found that with the appropriate persona setting and natural language prompts, LLMs can perform well on certain pragmatic language understanding tasks, suggesting that sociol-pragmatic methodologies can provide valuable insights into complex questions in LLMs’ pragmatic reasoning capacity assessment.

Along similar lines of research, Yue et al.^17^ studied manner implicatures as a part of pragmatic knowledge in LLMs, arguing that to become effective human-like social communicators, LLMs must be capable to handle the non-literal meanings of utterances. Yue et al.^17^ introduced *SwordsmanImp*, the first Chinese multi-turn dialogue dataset specifically designed to explore conversational implicature. This dataset is derived from dialogues in the Chinese sitcom *My Own Swordsman* and contains 200 meticulously crafted questions, each annotated with the specific Gricean maxims that are violated. Yue et al.^17^ evaluated eight LLMs, both closed-source and open-source, on two tasks: a multiple-choice question task and an implicature explanation task. Their findings show that GPT-4 achieves human-level accuracy (94%) on multiple-choice questions, with causal LLM following at 78.5%. Other models, including GPT-3.5 and several open-source models, perform worse, with accuracy ranging from 20% to 60% on these questions. Human raters assessed the LLMs’ explanations of implicatures based on reasoning ability, logic, and fluency. Although all models produced largely fluent and coherent text, only GPT-4 scored well on reasoning ability, suggesting that most LLMs struggle to provide satisfactory explanations for conversational implicatures. Furthermore, Yue et al.^17^’s analysis suggests that LLMs’ performance does not significantly differ across various Gricean maxims, indicating that they do not seem to process implicatures derived from different maxims differently.

Relatedly, comprehensive comparisons were conducted of human and several LLMs on pragmatic language understanding^18^. As laid out in Hu et al.^18^, pragmatics and the understanding of non-literal language are crucial aspects of human communication and have posed a long-standing challenge for artificial LLMs. They conducted a detailed comparison of LLMs and humans across seven pragmatic phenomena, using zero-shot prompting with a carefully curated set of English materials. Hu et al.^18^ investigated whether models choose pragmatic interpretations of speaker utterances, display similar error patterns to humans, and utilize similar linguistic cues as humans when solving these tasks. Their findings reveal that the largest models not only achieve high accuracy but also replicate human error patterns: when incorrect, LLMs tend to prefer literal interpretations over heuristic-based distractors. Additionally, they find initial evidence that both LLMs and humans are sensitive to similar linguistic cues. These results suggest that pragmatic behaviors can emerge in models without needing explicitly constructed representations of mental states. However, their results also indicate that LLMs often struggle with phenomena that depend on violations of social expectations.

While there has been considerable progress in evaluating LLMs’ understanding of pragmatic phenomena, previous studies have several notable limitations. Firstly, most existing research has concentrated on scalar and quantity implicatures or presuppositions, without giving sufficient attention to other types of implicatures, for example, manner implicatures, a key component of pragmatic reasoning that involves understanding why a speaker chooses a more complex expression over a simpler alternative. This omission limits our understanding of how LLMs interpret different kinds of context-sensitive language and manage various subtleties in communication that go beyond mere semantic content. Secondly, the currently available benchmarks and datasets, such as IMPPRES^4^ and SwordsmanImp^17^, are not designed to test LLMs on the full range of manner implicature inferences, namely those requiring sensitivity to the manner of expression. As a result, the existing literature do not fully capture the LLMs’ ability to process and generate language that reflects the intricate interplay between syntax, semantics, and a broader spectrum of pragmatics.

Furthermore, studies such as those conducted by Yue et al.^17^ and Cong^7^ provide valuable insights into LLM performance on specific metrics, such as using prompt engineering and generated text. Nevertheless, previous studies often do not explore the broader question of how well LLMs can generalize their performance to novel, varied metrics, such as those derived from probabilities or embeddings. This focus on narrow task- and metric-specific evaluations fails to address whether LLMs can adapt to different conversational scenarios that require a nuanced understanding of pragmatic cues. Additionally, while some research, such as Hu et al.^18^, has compared human and model performance on pragmatic language tasks, these studies typically do not investigate the mechanisms by which LLMs process pragmatic information or the extent to which these models rely on heuristic versus genuinely inferential reasoning. The lack of metrics variety might lead to the lack of exploration into the underlying cognitive processes of LLMs. Consequentially, this would limit our ability to explain, understand, and improve LLMs pragmatic capabilities. Finally, existing studies comparing contextual and static embeddings could greatly benefit from the current work, which attempts to rigorously examine these embeddings across a range of pragmatic tasks^19,20^. The absence of thorough, comparative studies examining different types of embeddings in handling Gricean pragmatic reasoning means that our understanding of LLMs’ true capabilities and limitations remains incomplete.

In summary, as far as our knowledge goes, there have been no systematic works examining the behavior of LLMs for manner implicatures, a central member of the class of context-sensitive phenomena^2,3,7^. These gaps highlight the need for more comprehensive research that includes manner implicatures and thorough metrics, leverages diverse datasets and benchmarks, and examines the cognitive processes of LLMs in greater detail. The current work attempts to address these limitations, providing an approach to better evaluate and enhance the pragmatic competence in LLMs.

### Manner implicatures in formal linguistics

We focused on manner implicatures triggered by three different linguistic elements, which have been introduced by Horn (1991)^1^, and have been widely studied empirically and theoretically in formal semantics and pragmatics^2,3,21^. First, we examined manner implicatures triggered by markedness-based negation. Markedness is essentially about Gricean maxim of manner. In a context in which two sentences are semantically equivalent, markedness is a measure of relative cost and complexity^2,3,22^. For example, by saying *I was not unaware of the problem*, a human speaker typically implies **(a)** or **(b)**^1^.

- Inference (a): *I was damn well aware of it.*
- Inference (b): *I had a very slight awareness of the problem.*

This is because despite being logically equivalent, the utterance with the negated negative antonym (NNA) *not unaware* is longer, more complex and costly, and more “marked” than *aware*, an alternative the speaker could have used but chose not to. Why not? One likely explanation is that the speaker intended to go beyond the literal semantics and convey pragmatically enriched meanings such as Inferences (a,b). Human speaker’s reasoning about “markedness” and their speech act of using the utterance instead of its alternative give rise to manner implicature inferences (a,b).

Apart from markedness NNA, we investigated manner implicatures triggered by causal constructions and modal verbs^2,22,23^. For causals, consider the utterance “*The intruders caused the victims to die*”. This sentence can have a literal interpretation where the intruders are understood to have directly killed the victims. However, the implied interpretation suggests a more indirect causation, such as arranged events that led to the deaths without direct involvement. The usage of causals adds syntactic complexity to the utterance. The manner implicature arises because the utterance is less straightforward and more costly in manner than the alternative that the speaker could have used but chose not to, “*The intruders killed the victims*”, which directly attributes the action to the intruders without implying any indirectness. Here, the intended interpretation of the utterance is the implied meaning highlighting the indirect causality. For modals, consider the utterance “*I had the ability to fix it*”, a listener computes a literal interpretation that the speaker could have fixed the problem and did so. However, the listener also infers an implied interpretation where the speaker had the ability but chose not to act. The manner implicature is drawn from the context and the choice of modal expression, suggesting that not acting was a deliberate decision. This contrasts with the more straightforward and less costly alternative, “*I was able to fix it*”, where no implicature is suggested, simply stating the action was within the speaker’s capability and was completed. In this case, the intended interpretation of the utterance is the implied reading emphasizing the speaker’s deliberate inaction despite having the capability. Causals and modals can trigger manner implicatures, changing the manner of the expression, hence adding depth and nuance to the listener’s understanding beyond the literal meaning^2,3^.

In addition to capturing manner implicature through the lens of its linguistic triggers, manner implicatures can be further characterized from the aspect of sentence relations. Formal semantics proposes that sentence relations such as entailment, implication, and neutrality are crucial in understanding different layers of meaning^1,3,24^. Entailment refers to a strict relationship where the truth of one sentence (the entailment) necessarily follows from the truth of another. Entailment is considered a tighter and more rigid relation than implicature, which concerns what is implied by an utterance. Implicatures, often derived from conversational context and Gricean maxims, do not have the same logical necessity as entailments. An utterance can imply another without entailing it, meaning the implied inference can be canceled or contextually dependent, whereas entailments cannot be easily dismissed without contradiction. Additionally, neutral relations indicate that utterances neither entail nor imply each other, showing no direct inferential connection between them. Driven by observations made in formal linguistics, we propose that a key indicator of understanding manner implicatures in LLMs is the success in teasing apart implications from other sentence relations such as entailment and neutral, showing robust sensitivity to different shades of meaning.

### Current work

Against the background of LLMs assessment and formal linguistics, the current work hopes to explain and demystify LLMs’ competence in manner implicatures. The current work categorizes metrics based on the levels at which they function within the LLMs: probabilities, embeddings, and generated text. We developed surprisal metrics based on negative log-probabilities, calculated (cosine) similarities of embeddings, and crafted natural language prompts to probe LLMs. Processing for all the metrics in this paper was conducted on a Purdue Compute Server with one NVIDIA Gilbreth A100 80GB GPU (https://www.rcac.purdue.edu/compute/gilbreth).

Specifically, we tested surprisal scores on manner implicatures triggered by causals and modals. Surprisals mathematically represent the negative log-probability of a word sequence given contexts as calculated by LLMs^25,26^. Conceptually, as a well-studied metric in computational psycho-linguistics, surprisals reflect how unexpected and surprising a sequence is, given the (preceding) context^26–31^. We hypothesized that if LLMs can represent and interpret manner implicatures in a way that is explainable under formal linguistics frameworks, they should assign a lower surprisal to a sequence that sounds natural and appropriate pragmatically and a higher surprisal otherwise.

The operation on (cosine) similarities of embedding vectors was applied to the manner implicatures triggered by NNA. Inspired by formal as well as distributional semantics^32–34^, we hypothesized that if LLMs have knowledge about pragmatically enriched meaning, in a (multi-dimensional) semantic space, different shades of meaning in language use should be represented by different degrees of semantic relatedness. We tested this hypothesis with contextual and static language models.

Besides direct probing methods such as deriving surprisals from LLMs token-wise log-probability and similarities from LLMs-embedding vectors, we probed LLMs to generate natural language text, as an indirect interpretation of LLMs’ behavior^29,35^. Using prompt engineering and LLMs-generated natural response to the curated prompts, we constructed multi-class and binary-class classification tasks. Our prediction is that if LLMs showed competence in manner implicatures, they should be able to classify different sentence relations in a pattern that is explainable and aligned with formal and distributional linguistic observations.

## Datasets creation and selection

The datasets consist of two parts: first, the **target** datasets, which include manner implicatures triggered by causal, modal, and negation; and second, the **baseline** datasets that contain sentence pairs with neutral and scalar implicatures relations, which are more widely studied than manner implicatures in LLMs explainability works.

Concretely, first, we constructed a **target** dataset for manner implicatures triggered by modal and causal, and one by negation. For the **target** dataset containing modal and causal, we expanded a small published experimental pragmatics stimuli set^23^ using prompt engineering. There were one set of manner implicature triggered by causality and two triggered by modality in the original dataset^23^. We prompted ChatGPT through OpenAI API (https://platform.openai.com/docs/api-reference/chat)^36^, using the following persona and instruction setting to obtain the implied interpretation^7^: “You are a very sociable person with a strong sense of pragmatic understanding. Please give a one-sentence interpretation of the sentence:”; and the following to obtain the literal interpretation^7^: “You are a nerd who can only get the literal meaning of an utterance. Please give a one-sentence interpretation of the sentence:”.

All the ChatGPT-generated synthetic stimuli were reviewed by two human raters: the author, a linguist, and an independent student scholar whose first language is American English. This review was conducted to ensure both acceptability and naturalness as well as alignment with the intended interpretation. For stimuli rating, we used a 1-7 Likert scale, where 1 indicates “unacceptable” and 7 indicates “completely acceptable”. Raters were instructed to consider how they would personally respond to hearing the sentence in natural conversation, rating the extent to which they agreed with its intended interpretation within the given context. The Likert scale captured varying degrees of agreement or acceptability, with intermediate values reflecting partial agreement. Specifically, for stimuli involving causal-type manner implicatures, the average acceptability rating was 6.25, with an agreement with the intended interpretation at 6.15. For manner implicatures triggered by modality, the stimuli averaged 7 in acceptability and 6.96 in agreement with the intended interpretation. The quality check of the synthetic data indicated that it is generally robust and validated, with most entries being acceptable. The intended interpretations align appropriately with contextual expectations, confirming that the data mostly reads as intended within the given contexts. Furthermore, crucially, our stimuli templates are rooted in the methodology of Wilson and Katsos (2016)^23^, whose empirical evidence substantiates the sensitivity to intended readings, providing further validation for our stimuli and experiment design.

Specific examples were given in Table 1. The **Utterance** in Table 1 means the actual sentence uttered by the speaker. The **Alternative** in Table 1 refers to a simpler plain sentence that the speaker reason about and could have used but chose not to. The hypothesis is that, when choosing to use the utterance, the speaker intended to convey an implied meaning that goes beyond the literal meaning; whereas when choosing to use the alternative, the speaker intended to convey the literal meaning^23^. After automatic prompting and manual checking, we curated 192 synthetic data triples in the format of *Sentence*, *Interpretation1*, and *Interpretation2* (i.e., 96 pairs of utterance and alternatives) for the causal-triggered manner implicatures, and 246 such triples (123 pairs) for the modality-triggered manner implicatures in total.

**Table 1:** Sample items from the **target** dataset of manner implicatures triggered by causal and modality.

Manner implicature trigger	Pair label	Sentence	Reading1 (literal)	Reading2 (implied)	Intended
Causal	Utterance	The intruders caused the victims to die	The intruders killed the victims directly	The intruders killed the victims indirectly	Reading2
	Alternative	The intruders killed the victims	The intruders killed the victims directly	The intruders killed the victims indirectly	Reading1
Modal	Utterance	I had the ability to fix it	I could have fixed it (and I did)	I could have fixed it but I didn’t	Reading2
	Alternative	I was able to fix it	I could have fixed it (and I did)	I could have fixed it but I didn’t	Reading1

Further, for the **target** dataset of manner implicatures triggered by negative antonyms, from the British National Corpus (BNC) written data^37^, we extracted sentences that contain adjectives used in a previous work^21^, which studied the distributional vector representations of NNAs for 184 pairs of English adjectives. We utilized Stanza^38^ to parse the BNC data. We focused on predicate adjectives, thus a *semgrex* rule with the form $[\{pos:JJ\}$>$cop (\{\})]$ was used to further isolate predicate adjectives from attributive adjectives. Further, we automatically substituted adjectives with their negative antonym pairs, and inserted modification strings *not*, *slightly*, or *damn well* in the appropriate positions. We chose such modifications because they have been examined in previous work^21^. The antonyms were generated using NLTK corpus reader *wordnet.synsets*^39,40^.

Given that certain sentences might no longer sound natural after the insertion of modifiers, we additionally conducted a text classification task using T5-large language model^41^ to tag sentence acceptability. Also, sequence length might influence LLMs’ behavior, thus we controlled sentence length and included only *short* sentences which have fewer than 21 words. About 50% of the sentences were discarded as they were unacceptable or overly lengthy after applying the automatic substitution and the sentence length filtering. Why half of the data were tossed? This is likely because negations with modal verbs or adverbials in the set of NNAs were often not amenable to such transformation. The position of negations causes anomaly. In total, there were 402 pairs of fully processed acceptable short sentences. As illustrated in Table 2, each pair consists of 4 sentence frames: Plain *I’m aware*; NNA *I’m* *not* *unaware*; Inference(a) *I’m* *slightly* *aware*; Inference(b) *I’m* *damn well* *aware*. Sample items of the **target** NNA-triggered manner implicatures were illustrated in Table 2.

**Table 2:** Sample items from the **target** dataset of manner implicatures triggered by markedness NNA.

Sentence1	Sentence2	Relations
I’m aware of it. [Plain]	I’m not unaware of it. [NNA]	Equivalent
I’m not unaware of it. [NNA]	I’m slightly aware of it. [Inference(a)]	Implied
I’m not unaware of it. [NNA]	I’m damn well aware of it. [Inference(b)]	Implied
I’m slightly aware of it. [Inference(a)]	I’m aware of it. [Plain]	Entailment
I’m damn well aware of it. [Inference(b)]	I’m aware of it. [Plain]	Entailment

Second, for the **baseline** datasets, we constructed two parts: (1) other cases of more widely studied implicatures to establish the comparison baseline: 1199 sets of *entailment* and *implied* sentence pairs were extracted from the widely cited scalar implicature dataset^4^, for which we focused on scalar implicature triggered by quantifiers; (2) *neutral* cases where sentences have no relations: 1094 sets of short sentences were extracted from the Stanford Natural Language Inference corpus (SNLI^42^). SNLI is a collection of 570,000 human-written English sentence pairs manually labeled for balanced classification. To ensure fair comparison, we only kept SNLI neutral sentences that have fewer than 21 words and whose annotator labels are 100% *neutral*, excluding cases with inter-rater disagreement. Sample items of **baseline** datasets were illustrated in Table 3.

**Table 3:** Sample items from the **baseline** datasets.

Sentence1	Sentence2	Relations
Some cashiers exercise	Not all cashiers exercise	Implied
All cashiers exercise	Some cashiers exercise	Entailment
Two men are standing in a boat	A few men are fishing on a boat	No inferential relation

## Surprisal-based metrics

### Experiment on surprisal

For the **target** dataset of manner implicatures triggered by causal and modality, we derived surprisal scores from LLMs for a sentence and its possible interpretation in a pair. We calculated LLMs-surprisals based on *minicons*^26^, a utility for behavioral and representational analyses of LLMs. We designed two surprisals related tests to examine how manner implicatures are represented and interpreted in LLMs. For both tests, we provided a brief context before the Sentence and a transitional question to bridge the Sentence and the interpretations. The context was typically one sentence long, generated using ChatGPT prompt engineering and validated manually. The transitional question was always “What does the speaker intend to convey?”. We included this explicit prompt to explore the speaker’s (implicit) intentions, considering that pragmatically enriched inferences–such as implicatures–are context-sensitive and can be canceled, unlike literal meanings. These implied meanings are not strictly truth-conditional and may not naturally emerge without such explicit prompts. Therefore, we used this prompt format to encourage the implied interpretation. Previous studies^7^ have shown that humans similarly respond to prompts designed in this way.

Utilizing *minicons*^26^, we took token-wise surprisals mean for the entire string combing context, *Sentence* (Utterance and Alternative as in Table 1), question, and *Reading* (Reading1 and Reading2 as in Table 1). Crucially, LLM-surprisal was calculated by summing the surprisals of individual tokens and then dividing by the sequence length. Longer sequences do not necessarily lead to higher surprisal values; in other words, the surprisal scores were normalized by length. Moreover, recall that when creating the datasets, we took into account sequence length, aiming to control for this variable as much as possible while maintaining the clarity of the interpretations.

Concretely, for the initial test, we hypothesized that LLMs-surprisals can serve as a proxy to the appropriateness of a sequence, and if LLMs show pragmatic sensitivity, they should assign a lower surprisal score to the intended meaning, which is associated with an appropriate natural language usage. Accordingly, we assigned 1 if a lower surprisal was generated for the intended reading sequence and 0 otherwise (c.f. Table 1). For example, given a *Sentence* “The intruders caused the victims to die”, LLMs were considered successful if they assigned a lower aggregated surprisal score to the sequence containing the intended reading “The intruders killed the victims indirectly” than the other reading.

For the critical tests, we assigned 1 only if LLMs’ showed explainable behavior for both sequences in a pair: they accurately gave a lower surprisal to the sequence containing the intended interpretation in both the *Utterance* and *Alternative* scenario in a pair (c.f. Table 1). For instance, a LLM was considered to have successfully represented manner implicatures if it gave a lower surprisal to the intended reading “The intruders killed the victims indirectly” than the other reading given the *Utterance sentence* “The intruders caused the victims to die”, and it gave a lower surprisal to “The intruders killed the victims directly” given the *Alternative sentence* “The intruders killed the victims”.

The LLMs success evaluation mechanisms in the initial and critical tests were summarized below. Note for the initial test, the chance level is set at 0.5, as there are two possible scenarios: either a lower surprisal is generated for the intended Reading (indicating success), or it is not (indicating failure), given the Sentence. On the other hand, for the critical test, there are four possible scenarios as illustrated in Table 4, and the chance level is 0.25. LLM is considered successful and accurate in the critical tests only for Scenario 2.

- Success in the initial test [chance level = 0.5]:
  *tokenwise* *Surprisal* *mean*(Intended Reading | Sentence) <
  *tokenwise* *Surprisal* *mean*(The other Reading | Sentence)
- Success in the critical test [chance level = 0.25]:
  *tokenwise* *Surprisal* *mean*(Reading2 | Utterance) <
  *tokenwise* *Surprisal* *mean*(Reading1 | Utterance) and
  *tokenwise* *Surprisal* *mean*(Reading1 | Alternative) <
  *tokenwise* *Surprisal* *mean*(Reading2 | Alternative)

**Table 4:** A summary of the four possible scenarios illustrating LLM behaviors in the critical tests.

Scenario	Tokenwise LLM-surprisal mean	Accurate
1	$Surprisal\_mean(Reading2 | Utterance) < Surprisal\_mean(Reading1 | Utterance)$, $Surprisal\_mean(Reading2 | Alternative) < Surprisal\_mean(Reading1 | Alternative)$	0
2	$Surprisal\_mean(Reading2 | Utterance) < Surprisal\_mean(Reading1 | Utterance)$, $Surprisal\_mean(Reading1 | Alternative) < Surprisal\_mean(Reading2 | Alternative)$	1
3	$Surprisal\_mean(Reading1 | Utterance) < Surprisal\_mean(Reading2 | Utterance)$, $Surprisal\_mean(Reading2 | Alternative) < Surprisal\_mean(Reading1 | Alternative)$	0
4	$Surprisal\_mean(Reading1 | Utterance) < Surprisal\_mean(Reading2 | Utterance)$, $Surprisal\_mean(Reading1 | Alternative) < Surprisal\_mean(Reading2 | Alternative)$	0

We ran both the initial and critical tests on the 192 sets of causal-triggered manner implicatures and the 246 sets of modality-triggered manner implicatures. The following LLMs were selected for the surprisal-based metrics: a encoder-only masked language model *FacebookAI/roberta-large*^43^, and recent decoder-only causal language models including *EleutherAI/gpt-neo-1.3B*^44^, *meta-llama/Llama-2-7b-hf*^45^, *tiiuae/falcon-7b*^46^, *mosaicml/mpt-7b*^47^, *mistralai/Mistral-7B-v0.1*^48^, and *Qwen1.5-MoE-A2.7B*^49^. Specifically, Llama2 is a recent LLM developed by Meta, featuring improved training efficiency and performance over its predecessors. GPT-Neo, developed by EleutherAI, is an open-source alternative to GPT-3 that aims to provide accessible, high-quality language modeling capabilities. Falcon-7B, developed by TII, is a language model with 7 billion parameters, trained on 1,500 billion tokens from RefinedWeb, supplemented with curated corpora. MPT-7B, created by MosaicML, is a decoder-style transformer model pre-trained from scratch on 1 trillion tokens of English text and code. This model is part of the Mosaic Pretrained Transformer (MPT) family, which features a modified transformer architecture designed for efficient training and inference. Mistral-7B-v0.1 is another decoder-based language model. Qwen1.5-MoE-A2.7B is a transformer-based decoder-only LLM that employs Mixture of Experts (MoE) architecture. All the LLMs are open-sourced and hosted in HuggingFace^30^ (https://huggingface.co/).

### Explain LLMs’ behavior via surprisals

To explain and understand LLMs’ representation of causal and modal verbs triggered manner implicatures, we presented surprisals-based metrics findings in Table 5. For the initial test, we computed how much time LLMs assigned a lower surprisal to the sequence containing the intended interpretation in the **target** datasets. Results suggested that most LLMs showed at chance or below chance accuracy. RoBERTa gave slightly above chance accuracy in the modality-triggered manner implicature dataset, and MPT gave slightly above chance accuracy in the causal-triggered manner implicature dataset. Overall, our initial test findings indicated that LLMs’ representation and interpretation of manner implicatures were not robust, and their behavior was not consistently explainable under the formal semantics and pragmatics theory.

**Table 5:** Accuracy in the initial and critical tests for the **target** dataset of manner implicatures triggered by causal and modality. Notation: For Critical test, S1 refers to Scenario 1 as in Table 4, with S2 through S4 similarly denoting Scenarios 2 through 4. Accuracy (when LLMs fall into Scenario 2) is shown in bold.

Surprisal-based test	Implicature trigger	RoBERTa	GPTNeo	Llama	Falcon	MPT	Mistral	Qwen
Initial test	Causal	0.453	0.495	0.484	0.484	0.516	0.50	0.523
Initial test	Modality	0.598	0.50	0.50	0.50	0.50	0.50	0.50
Critical test	Causal	S1 0.698, **S2 0.062**, S3 0.156, S4 0.083	S1 0.99, **S2 0.00**, S3 0.01, S4 0.00	S1 0.010, **S2 0.00**, S3 0.031, S4 0.958	S1 0.948, **S2 0.010**, S3 0.042, S4 0.00	S1 0.885, **S2 0.062**, S3 0.031, S4 0.021	S1 0.010, **S2 0.010**, S3 0.010, S4 0.969	S1 0.958, **S2 0.042**, S3 0.00, S4 0.00
Critical test	Modality	S1 0.667, **S2 0.211**, S3 0.016, S4 0.106	S1 0.00, **S2 0.00**, S3 0.00, S4 1.0	S1 0.00, **S2 0.00**, S3 0.00, S4 1.0	S1 0.00, **S2 0.00**, S3 0.00, S4 1.0	S1 0.00, **S2 0.00**, S3 0.00, S4 1.0	S1 0.00, **S2 0.00**, S3 0.00, S4 1.0	S1 0.00, **S2 0.00**, S3 0.00, S4 1.0

For the critical test, we computed how much time LLMs assigned a lower surprisal to the intended interpretation for both sentences in a pair, namely Scenario 2 as in Table 4. As shown in Table 5, most LLMs showed zero or close to zero accuracy. Among all the LLMs, RoBERTa showed the best performance on the modality-triggered manner implicature dataset, successfully falling into Scenario 2 in 21.1% of cases. The other LLMs dominantly and indistinguishably fell into Scenario 4 or Scenario 1, where they insensitively assigned a lower surprisal to the same Reading, regardless of whether the given input was the Utterance or the Alternative. To sum up, critical tests on surprisal-based metrics suggested that LLMs did not show sophisticated understanding of manner implicatures, and more recent and larger LLMs did not lead to strikingly better behavior.

**Table 6:** Accuracy, without explicit prompting, in the initial and critical tests for the **target** dataset of manner implicatures triggered by causal and modality. Notation: For Critical test, S1 refers to Scenario 1 as in Table 4, with S2 through S4 similarly denoting Scenarios 2 through 4. Accuracy (when LLMs fall into Scenario 2) is shown in bold.

Surprisal-based test	Implicature trigger	RoBERTa	GPTNeo	Llama	Falcon	MPT	Mistral	Qwen
Initial test	Causal	0.484	0.495	0.50	0.505	0.510	0.49	0.484
Initial test	Modality	0.736	0.50	0.50	0.50	0.50	0.50	0.50
Critical test	Causal	S1 0.438, **S2 0.146**, S3 0.177, S4 0.240	S1 0.99, **S2 0.00**, S3 0.01, S4 0.00	S1 0.010, **S2 0.00**, S3 0.021, S4 0.969	S1 0.927, **S2 0.021**, S3 0.010, S4 0.042	S1 0.979, **S2 0.021**, S3 0.00, S4 0.00	S1 0.010, **S2 0.00**, S3 0.021, S4 0.969	S1 0.969, **S2 0.00**, S3 0.031, S4 0.00
Critical test	Modality	S1 0.179, **S2 0.488**, S3 0.016, S4 0.317	S1 0.00, **S2 0.00**, S3 0.00, S4 1.0	S1 0.00, **S2 0.00**, S3 0.00, S4 1.0	S1 0.00, **S2 0.00**, S3 0.008, S4 0.992	S1 0.00, **S2 0.00**, S3 0.00, S4 1.0	S1 0.00, **S2 0.00**, S3 0.00, S4 1.0	S1 0.00, **S2 0.008**, S3 0.00, S4 0.992

Since humans can often infer implicatures without being explicitly prompted about the speaker’s intent, we may not expect LLMs to reason accurately in this relatively unnaturalistic format of overtly probing speaker’s intent. To address this potential confound, we reproduced the computations without the prompt “What does the speaker intend to convey?”. As shown in Table 6, the results were mostly consistent with those in Table 5 (with explicit prompting). RoBERTa showed the most notable change, with accuracies in critical tests increasing and accuracy in the initial test on modality-type implicatures also improving. The other LLMs remained largely insensitive to minimal differences in input, indiscriminately favoring a particular Reading without distinguishing between input Sentence.

## Similarity-based metrics

### Experiment on similarity

To characterize LLM explainability from the aspects of sentence relations and distributional relatedness, for the **target** dataset of manner implicatures triggered by markedness NNA and the **baseline** dataset, we embedded sequences with contextual and static embedding vectors. We started with the standard representational similarity measure–cosine similarity–to understand how LLMs represent different shades of meaning.

Apart from the widely used method of cosine similarities, as a supplementary analysis, we computed non-parametric rank-based Spearman correlation coefficients of the embedding vectors. Study has shown that as a more robust metric, Spearman is not affected by the rogue dimensions of contextual LLMs^33,50^. This rank-based approach has been illustrated to be particularly useful when outliers–such as rogue dimensions in contextualized language models–might otherwise distort traditional similarity measures such as cosine similarity^50,51^. Rogue dimensions in contextualized embeddings tend to disproportionately influence the overall similarity measure, particularly in models such as RoBERTa and GPT-2^33,50^. These dimensions dominate cosine similarity calculations because of their extreme variance. Crucially, Spearman’s $\rho$ is robust to such outliers and can serve as a form of postprocessing for embedding similarity: Spearman correlation transforms the embedding vectors into their ranks, effectively reducing the influence of rogue dimensions without requiring computation over the entire corpus or removing top principal components.

To summarize, we used two methods for similarity-based comparisons: standard cosine similarity, and Spearman correlation coefficients between embedding vectors. This combination of methods was intended to strengthen our analysis by capturing linguistic relationships through multiple robust similarity measures. Specifically, our linking hypothesis bridging formal and distributional linguistics, in particular, similarity-based metrics is as follows. The most related sentences should be in a tighter relation, with equivalence being a more strict and closer relation than entailment, followed by implications, particularly, manner implicatures, and finally the neutral, which indicates no inferential relations. Translated into distributional semantics, vector representations of sentences with close semantic relation (*equivalence*) should have the highest degree of relatedness and similarity, followed by *entailment*, whereas those that are *implied* should have a low degree of relatedness and similarity, but it is still higher than the *neutral*. Hence, we propose the following:

- The ranking of relatedness:
  *Equivalence* > *Entailment* > *Implication* > *Neutral*

The hypothesis leads us to predict that, if LLMs are able to represent and distinguish different shades of meaning in use, we should observe the following: (1) semantic vectors representing Plain utterances (e.g. *I’m* *aware* *of it*) and NNA should be the most semantically related since they represent semantic *equivalence*; (2) Similarly strong relatedness should be observed in the *entailment* relation of Plain and Inferences(a,b); (3) NNA *implies* Inferences(a,b), thus they should have weak relations and show low similarity. (4) Vectors of *neutral* inferences should show the lowest relatedness, hence the lowest similarity.

We computed similarity-based metrics for the following LLMs: contextual LLMs stsb-RoBERTa-large^52,53^, sentence-T5-base^41,54^, GPT-3 *text-embedding-ada-002*^55^, Gemini-flash-1.5^56^, and static GloVe^34^. We additionally conducted Wilcoxon rank-sum pairwise tests for the comparison of medians in two groups of non-parametric data, in order to examine if the the location of medians in two independent samples are statistically significant. Such pairwise tests were used to suggest and visualize if there was any statistical significance between LLMs’ representations of different kinds of sentence relations.

### Explain LLMs’ behavior via similarity

To explain and understand LLMs’ representation of markedness NNA triggered manner implicatures, we presented similarity-based metrics results. For the **baseline** dataset, cosine similarities mean and Spearman correlation coefficients mean were computed (Table 7). All models showed the lowest similarities and coefficients mean for embeddings of sentences with *neutral* relations, all but T5 showed the highest mean for *entailment*, with the *implication* fall in between. This is especially striking in RoBERTa. Our findings in the **baseline** dataset established our interpretation baseline. It validated our hypothesis about the ranked similarity and relatedness: *entailed* > *implied* > *neutral*. Additionally, we found no critical difference between static embeddings from GloVe and contextual ones from LLMs.

**Table 7:** Cosine similarity mean and Spearman correlation coefficients *Rho* mean in the **baseline** dataset.

Relations	GloVe		T5		RoBERTa		GPT-3		Gemini	
	Cosine	*Rho*	Cosine	*Rho*	Cosine	*Rho*	Cosine	*Rho*	Cosine	*Rho*
Neutral	0.83	0.67	0.85	0.85	0.47	0.45	0.89	0.74	0.61	0.59
Entailment	0.96	0.93	0.93	0.92	0.81	0.79	0.96	0.91	0.73	0.72
Implication	0.95	0.91	0.94	0.93	0.75	0.73	0.95	0.89	0.73	0.71

The **target** dataset cosine similarities mean and the corresponding Spearman correlation coefficients mean were given in Table 8. We interpreted the values in *neutral* and *equivalent* as the range, representing a hypothetical semantic space where *neutral* leads to the lowest similarity and *equivalent* the highest. Our findings suggested that models’ representation of the **target** manner implicature was not as interpretable as the **baseline**. Suppose that ’neutral’ and ’equivalent’ form the range, representing the lowest and highest boundary points of a semantic space, aligning with our hypothesis about semantic space and the ordering of similarity among different semantic relations, all models gave the lowest mean in the *neutral*. However, no models showed the highest mean scores in *equivalent*, and all but Gemini showed higher mean in *entailment* than in *implied*. Our findings indicated that models can tease apart *neutral* from *entailment* and *implication*, and most models showed consistency in distinguishing subtler relations such as *entailment* from *implied*. All models failed in ranking *equivalence* with the highest similarity. Interestingly, the recent LLM Gemini-flash-1.5 showed the highest similarity mean for one of the entailments (but not both) and not the *equivalent*, which was not interpretable under the current hypotheses. Notice that among all the models, metrics difference is the most salient for GPT-3: switching from standard cosine to Spearman showed observable difference, suggesting that rogue dimensions might affect representational quality in GPT-3^33,50^. There were no critical difference between GloVe and the LLMs, although we did find that the difference between entailment and implication appeared to be more observable in LLMs than in GloVe.

**Table 8:** Cosine similarity mean and Spearman correlation coefficients *Rho* mean for the **target** dataset of manner implicature triggered by markedness NNA.

Relations	GloVe		T5		RoBERTa		GPT-3		Gemini	
	Cosine	*Rho*	Cosine	*Rho*	Cosine	*Rho*	Cosine	*Rho*	Cosine	*Rho*
neutral	0.83	0.67	0.85	0.85	0.47	0.45	0.89	0.74	0.61	0.59
equivalent [Plain,NNA]	0.98	0.98	0.96	0.96	0.88	0.85	0.96	0.9	0.78	0.76
entailed [Inference(a),Plain]	0.99	0.99	0.97	0.97	0.94	0.92	0.97	0.94	0.95	0.94
entailed [Inference(b),Plain]	0.98	0.98	0.98	0.98	0.95	0.93	0.97	0.93	0.74	0.72
implied [NNA,Inference(a)]	0.97	0.97	0.96	0.95	0.88	0.87	0.97	0.92	0.80	0.78
implied [NNA,Inference(b)]	0.97	0.97	0.94	0.94	0.86	0.85	0.96	0.91	0.88	0.87

**Fig. 1:**
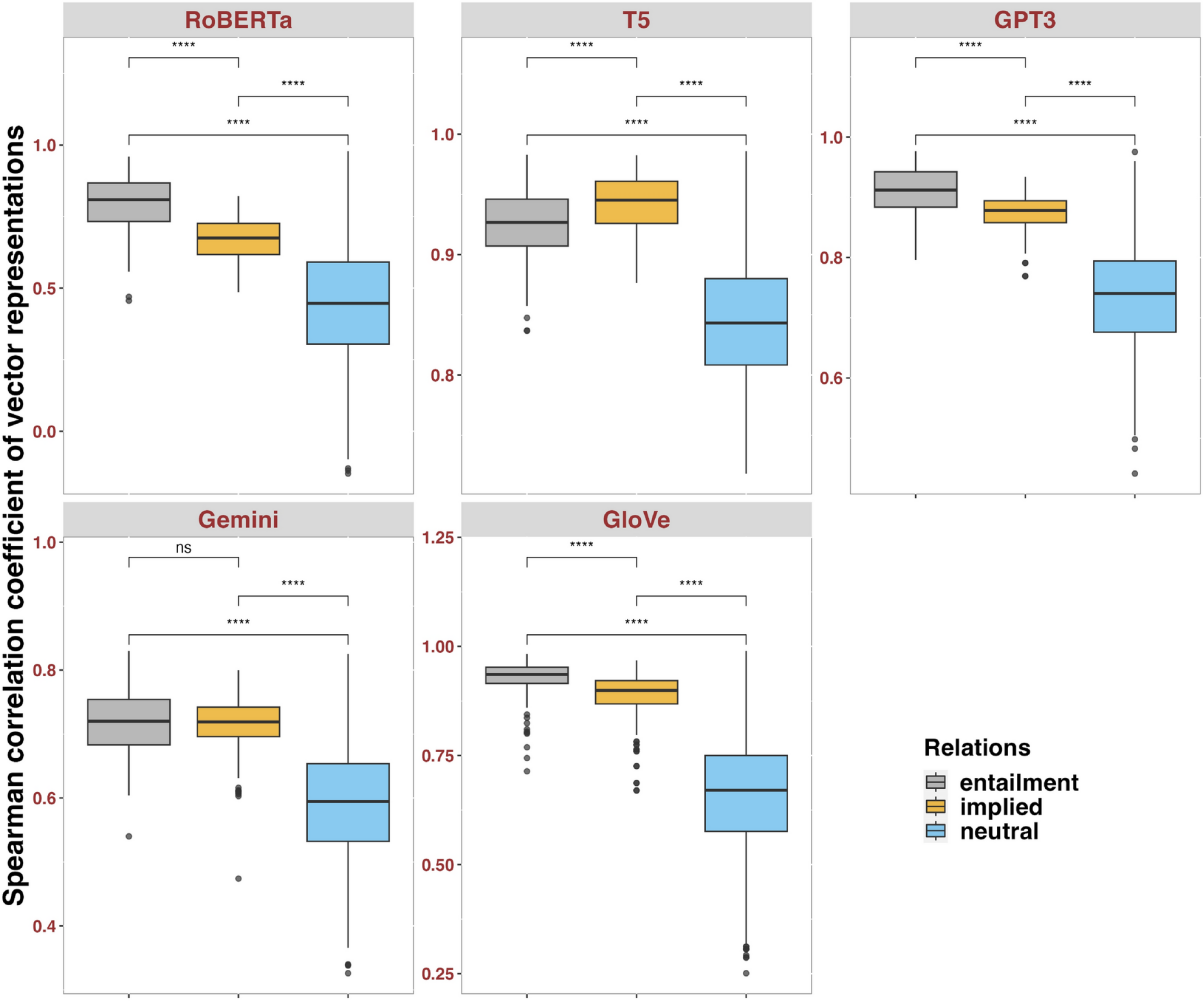
Wilcoxon tests with Bonferroni correction, comparing Spearman correlation coefficients of embedding vectors for the **baseline** dataset. Notation: ns: *p*>0.05; *: *p*≤0.05; ** *p*≤0.01; *** *p*≤0.001; **** *p*≤0.0001.

Further, we examined whether the difference in models’ representations of different relations was statistical. Taking pairwise comparisons of Spearman correlation coefficients, we found significant group comparison effects across most models in the **baseline** dataset (Fig.1), suggesting that both contextual LLMs and GloVe can generally differentiate entailment, implied and neutral. Only Gemini showed null effects in distinguishing entailment and implied. Boxplots showed that RoBERTa, GPT-3 and GloVe more closely aligned with formal and distributional linguistics observations than T5, with sequence pairs’ embeddings in entailment relations showing high correlation coefficients, hence close relatedness, neutral showing low coefficients, hence moderate relatedness, and the implied in between. Contextual LLMs did not strikingly outperform the static GloVe. Box-plots also indicated large amounts of outliers in GloVe and relatively large variance when using RoBERTa, given its y-axis spanning approximately from 0.0 to 1.0.

**Fig. 2:**
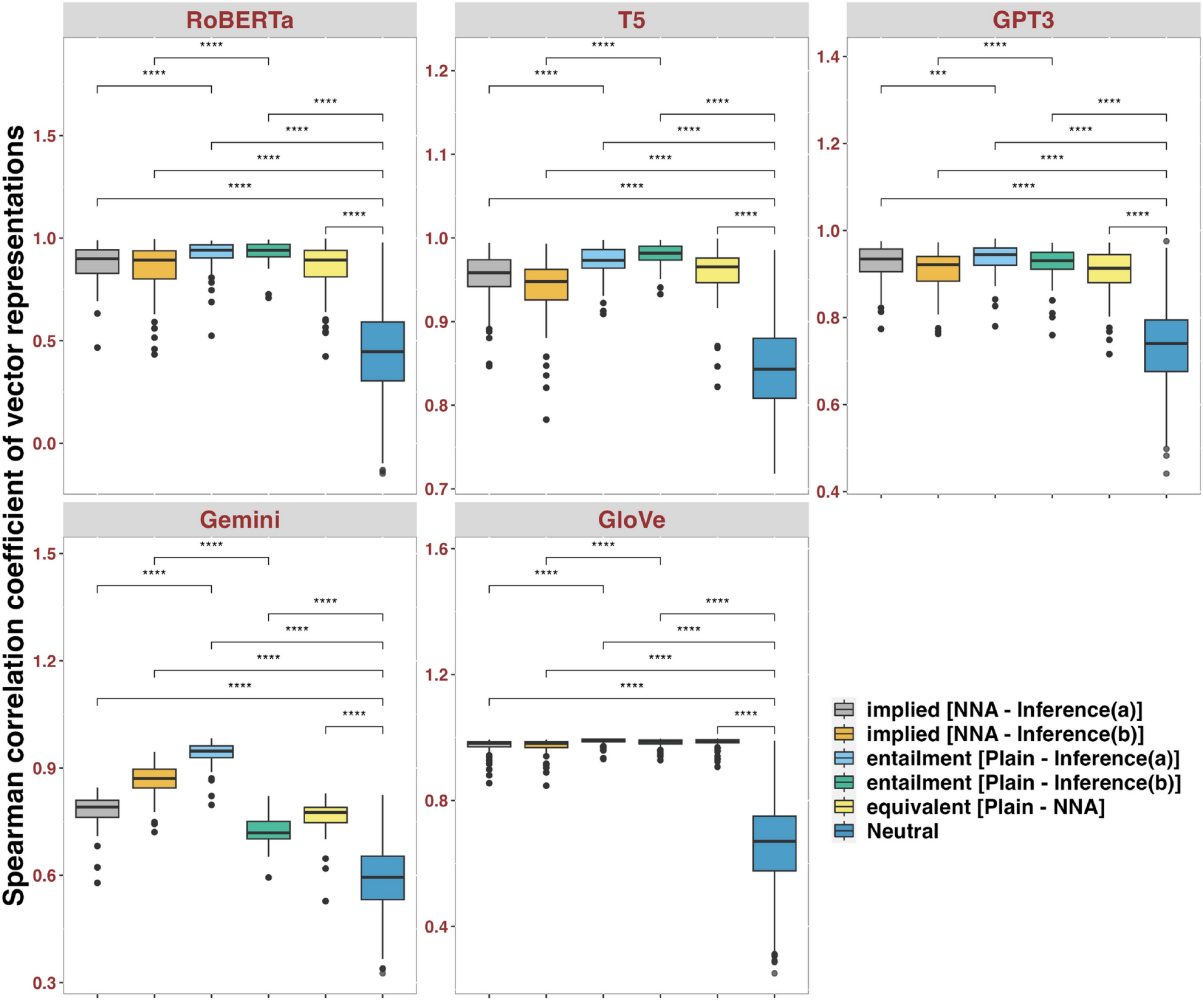
Wilcoxon tests with Bonferroni correction, comparing Spearman correlation coefficients of embedding vectors for the **target** manner implicature dataset. Notation: ns: *p*>0.05; *: *p*≤0.05; ** *p*≤0.01; *** *p*≤0.001; **** *p*≤0.0001.

In the **target** dataset (Fig. 2), pairwise Wilcox tests suggested that all models showed the weakest relatedness in *neutral*, which is in line with the formal and distributional linguistics predictions. For illustration purposes, although thorough statistical tests were conducted, only crucial pairwise comparisons were displayed. For example, comparisons between Inference(a) and Inference(b) were not illustrated in Fig. 2, since their relatedness is not directly informative of addressing our hypotheses. Results in Fig. 2 suggested that no models gave the full rank of closeness in the right direction (*Equivalence* > *Entailment* > *Implication* > *Neutral*), although most models showed statistical sensitivity to the distinction of equivalence, entailment, implication, and neutral. Gemini showed divergence for the entailment relation between Plain and Inference(b), which gave surprisingly low coefficients. This is unexpected under the current hypotheses. Contextual LLMs did not show strikingly better performance than static GloVe in detecting manner implicatures. All models have many outliers, with RoBERTa showing the largest amount of variance since its y-axis spanning approximately from 0.0 to 1.5. Statistical tests also indicated that RoBERTa showed null effects in distinguishing implied from equivalence, and that GPT-3 showed no statistical competence of teasing apart manner implicature (NNA, Inference(b)) from semantic equivalence (Plain, NNA).

## Prompting-based metrics

### Experiment on prompting

To further strengthen our investigation, we conducted a supplementary analysis using prompt engineering. There are two primary motivations, First, the surprisal and similarity-based metrics are directly derived from LLMs’ probability distributions and embedding representations, while our third metric focuses on the indirect interpretation of LLM’s behavior. Taken together, we argue that these three types of metrics can cross-validate and strengthen our study. Another motivation for this prompting-based metric is to complement and further enhance the similarity-based metrics. Although our experiments did not aim to explore how LLMs represent directionality in such asymmetric relations as entailment, and we focused on the degree of closeness between relations and how effectively this difference can be captured within a multidimensional semantic space, it is still plausible that our similarity operation resulted in unexpectedly lower similarity for Inference(a) and Plain, not because LLMs misrepresent entailment relations, but because, although Inference(a) entails Plain, Plain neither entails nor implies Inference(a). To address the potential issue of “wrong for the wrong reason”, we computed prompting-based metrics.

Concretely, we conducted an additional analysis by prompting *Gemini-flash-1.5*^56^ and *GPT-4o-mini*^36^ to explicitly identify these relations. Gemini-flash-1.5 is a lightweight LLM designed to handle long contexts^56^, with a context window of over two million tokens, enabling it to process extensive inputs such as long instructions. We carefully controlled LLM’s input: the utterance pairs and their orders are strictly based on Table 2,3. For example, Inference(a) was always “Sentence1”, followed by Plain, which was always the second sentence “Sentence2”.

We constructed three tasks. First, multi-class classification task. We used the following prompt instruction to probe LLM: *There are four kinds of relations between two utterances: (1) logically equivalent (2) entail (3) implicate (4) no relations. Here is the difference between (2) entailment and (3) implicature: (2) Entailment refers to a relation where the truth of a guarantees the truth of b; and if b is false, then a is also necessarily false. Entailments are not cancelable! (3) Implicatures refer to a relation where the truth of a implies, but does not guarantee, the truth of b; and if b is false, a can still be true (though perhaps under-informative or misleading). Implicatures can be canceled!* ***Your task*** *is to choose a relation (1), (2), (3), or (4) to accurately classify the relations for the following utterance pairs*. For all the prompting tasks, we used LLMs APIs’ default parameters setting. The accuracy was then calculated based on how much LLM’s responses and the gold labels of relations match. The gold label of each relation was illustrated in Tables 2 and 3.

Second, for the binary classification **initial** task, we used the same instructions explaining the different kinds of relations in (1-4). For LLM generation task instruction, we used the following prompt *Your task is to judge whether the claimed relation for the following utterance pairs is True or False. Please only output 1 for True or 0 for False without explaining.*. The claimed relation is the relation predicted by formal and distributional linguistics theory (c.f., Table 2). Specifically, LLM was considered to have successfully interpreted manner implicatures if it assigned True to the following relations:

1. Plain$\rightarrow$NNA: equivalent
2. NNA$\rightarrow$Plain: equivalent
3. Inference(a)$\rightarrow$Plain: entail
4. Plain$\rightarrow$Inference(a): neutral
5. Inference(b)$\rightarrow$Plain: entail
6. Plain$\rightarrow$Inference(b): neutral
7. NNA$\rightarrow$Inference(a): imply
8. Inference(a)$\rightarrow$NNA: entail
9. NNA$\rightarrow$Inference(b): imply
10. Inference(b)$\rightarrow$NNA: entail

Third, for the binary classification **critical** task, the same prompting instructions were used. LLM was considered to have successfully interpreted manner implicatures if it assigned True to both sentences in a pair of (1,2), (3,4), (5,6), (7,8), and (9,10), as illustrated in the above list.

### Explain LLMs’ behavior via prompting

For the multi-class classification task, accuracy was calculated based on how much LLM’s responses and the gold labels aligned. We additionally reported the variation (standard deviation: SD) of LLM’s responses distribution. Our findings indicated that for this particular metric, Gemini’s interpretation of sentence relations concerning NNA-triggered manner implicatures was generally not in line with what has been established in formal semantics. The **target** dataset gave 0.28 accuracy, whereas the **baseline** dataset gave 0.48. Interestingly, there were more variation in Gemini’s response (SD = 0.50) to the **baseline** dataset than the **target** dataset of manner implicatures triggered by markedness NNA (SD = 0.17).

For the binary classification initial test, results were given in Table 9. Findings suggested that Gemini showed difficulty characterizing and classifying neutral relations. Interestingly, Gemini was able to identify the implication relation between NNA and Inference(a) but not the entailment relation when the sentence pair was swapped. Yet for the pair of NNA and Inference(b), Gemini’s accuracy showed the inverse. On the other hand, GPT-4o showed difficulty capturing equivalence. It also showed less robustness when characterizing the implication pairs of NNA and Inference(a,b) (0.241). Overall, the initial test revealed that the two LLMs showed very different limitations, Gemini was more unstable and gave zero or close to zero accuracy when classifying the same pair with the swapped order, and Gemini showed relatively robust performance in classifying the pair of Plain and NNA, whereas GPT-4o was good at classifying the pair of Inference(b) and Plain.

**Table 9:** Initial test accuracy of binary classification of different relations in Gemini-Flash and GPT-4o.

Sentence pair	Relations	Accuracy (Gemini-Flash)	Accuracy (GPT-4o)
Plain$\rightarrow$NNA	Equivalent	0.983	0.632
NNA$\rightarrow$Plain	Equivalent	0.983	0.361
Inference(a)$\rightarrow$Plain	Entail	0.833	0.408
Plain$\rightarrow$Inference(a)	Neutral	0.00	0.843
Inference(b)$\rightarrow$Plain	Entail	0.998	0.995
Plain$\rightarrow$Inference(b)	Neutral	0.00	0.868
NNA$\rightarrow$Inference(a)	Imply	0.831	0.241
Inference(a)$\rightarrow$NNA	Entail	0.460	0.547
NNA$\rightarrow$Inference(b)	Imply	0.398	0.241
Inference(b)$\rightarrow$NNA	Entail	0.990	0.684

For the binary classification critical test, results were given in Table 10. Gemini showed zero or close to zero success in characterizing the pair of Plain and Inference(a,b) and poor accuracy in identifying the relations between the pair of NNA and Inference(a,b). In contrast, GPT-4o showed poor accuracy for all the pairs but Plain and Inference(b). To sum up, the prompting-based metrics indicated that the recent LLMs did not show sophisticated representation or interpretation of manner implicatures triggered by markedness NNA, and their behavior was not as explainable as a formal semantics and pragmatics theory would be in capturing natural language understanding.

**Table 10:** Critical test accuracy of binary classification of different relations in Gemini-Flash and GPT-4o.

Sentence pair	Accuracy (Gemini-Flash)	Accuracy (GPT-4o)
Plain, NNA	0.983	0.316
Plain, Inference(a)	0.00	0.338
Plain, Inference(b)	0.00	0.863
NNA, Inference(a)	0.311	0.139
NNA, Inference(b)	0.398	0.182

## Discussions

### Causals and modals triggered manner implicatures in LLMs

The findings from the surprisal-based metrics reveal a significant gap in the capacity of LLMs to represent and interpret manner implicatures, as demonstrated in Table 5. The results indicate that most LLMs, including more recent and larger LLMs, performed at or below chance levels when tasked with interpreting manner implicatures triggered by causal and modality. This lack of sophistication suggests that these LLMs may not effectively capture the subtleties of implicature that require nuanced pragmatic reasoning beyond lexical semantics or syntactic cues. Even RoBERTa, which showed slightly better performance for modality-triggered implicatures, and MPT, which showed relatively better results for causal implicatures, only performed marginally above chance. Critical tests further reveal LLMs’ lack of robustness and systematic sensitivity to manner implicatures. This could indicate that while these LLMs might show some ability to detect specific patterns associated with implicature triggers, their overall understanding and reasoning remain shallow and unaligned with formal semantic and pragmatic theories.

Several factors may explain these findings. One possibility is that the training data for these LLMs does not provide enough examples of implicatures or does not emphasize the types of pragmatic inferences required to precisely interpret these cases. Implicatures, by their nature, are context-dependent and often subtle, implicit, and covert, making them harder for LLMs to learn from standard corpora that focus more on general language use rather than specific pragmatic phenomena. Moreover, the architectures of these LLMs are primarily designed for capturing surface-level patterns and may lack the deeper abstraction or inferential capabilities needed to handle pragmatic nuances effectively^4,7,8^. The below-chance accuracy in the critical test further emphasizes that current LLMs do not have a robust or reliable mechanism to conduct pragmatic reasoning, particularly when the reasoning derivations are not overtly or directly marked in natural language sequences.

The implications of these results are significant for the development and evaluation of LLMs, particularly in contexts where understanding subtle language use is critical, such as in human-computer interaction or advanced language translation. The current findings suggest that reliance on LLMs for tasks that require such sophisticated language understanding as interpreting implicatures, may be premature. To improve performance, future LLMs may need to incorporate more holistic representations that better capture the interplay between context, inference, and utterance, potentially by integrating more formal semantic and pragmatic frameworks into their training and evaluation processes^2,3,17,18^. Moreover, these results highlight the need for more targeted datasets that explicitly model the kinds of implicit, unspoken, context-dependent inferences involved in pragmatic language use, which could provide more informative training signals for LLMs^4,5,7^.

### Negation triggered manner implicatures in LLMs

Our findings on LLMs and markedness NNA triggered manner implicatures suggest significant implications for LLMs’ ability to differentiate different shades of meaning. The observation that models generally perform better in distinguishing *entailment* and *implied* relations from the *neutral* ones indicates that LLMs and GloVe embeddings are somewhat capable of reflecting semantic hierarchies, as hypothesized. However, the fact that the performance of some of these models is less consistent and robust in separating *entailment* from *implied* or *equivalent* relations raises concerns about the models’ sensitivity to more nuanced semantic distinctions. This could be an area for further refinement in LLMs, potentially through fine-tuning or incorporating more pragmatically enriched training data.

The lack of a critical difference between static embeddings (such as GloVe) and contextual embeddings (from LLMs) in these tasks challenges the common assumption that LLMs always provide superior semantic representations. Our results suggest that while LLMs might offer certain advantages, particularly in context-dependent tasks, they do not consistently outperform the classic, static methods such as GloVe when it comes to distinguishing fine-grained proposition relations^50^. This finding could influence how NLP practitioners choose and implement models depending on the specific task at hand. For example, tasks requiring the discrimination of subtle semantic differences might still benefit from using or at least combining static embeddings with LLMs, rather than relying solely on contextual models.

Furthermore, the variability and presence of outliers, particularly in models such as GPT-3 and RoBERTa, highlight potential instability in how these models process and represent semantics and pragmatics. The observable differences between metrics, such as cosine similarity and Spearman correlation, particularly in GPT-3, suggest that these models might have “rogue dimensions” that affect their overall representational quality. This points to the need for more robust evaluation and possibly the development of new techniques to stabilize and refine these embeddings. Overall, while LLMs show promise in capturing complex semantic relations, these findings emphasize the necessity for continued research and improvement to enhance their interpretability and reliability, especially in tasks requiring fine-grained semantic and pragmatic differentiation.

It is worth highlighting a crucial limitation in the use of symmetric similarity measures such as cosine similarity and Spearman coefficient for analyzing inherently asymmetric relations such as entailment and implicature. While our study focused on the degree of closeness between these relations within a multidimensional semantic space, it became evident that the symmetry of the similarity operations may have contributed to the unexpected results, particularly the surprisingly lower similarity of *entailment* relations between Inference(a,b) and Plain (c.f. GPT-3 and Gemini in Fig. 2). The issue lies in the fact that while Inference(a,b) entails Plain, the reverse is not true: Plain neither entails nor implies Inference(a,b). This asymmetry is critical in semantic and pragmatic analyses, suggesting that the similarity method, as currently applied, might not fully capture the directional nature of these relations. Therefore, the apparent misalignment between the expected and observed similarities could be attributed to the inherent limitations of symmetric measures rather than a fundamental misrepresentation by the LLMs.

### Prompting manner implicatures in LLMs

The findings from the prompting-based metrics, as outlined in the results, further reveal the challenges that LLMs face, as shown in Table 9 and Table 10. In the multi-class classification task, Gemini-Flash’s alignment with gold-standard labels was consistently below chance levels, especially for the **target** dataset of manner implicatures triggered by markedness NNA. This suggests that the model struggles to accurately classify these implicatures and that its internal representations likely do not align with established formal linguistic theories. The high variability in Gemini-Flash’s responses for the **baseline** dataset compared to the lower variability for the **target** dataset also indicates inconsistency in its interpretation mechanisms, further reflecting its lack of robustness in pragmatic reasoning and understanding.

In the binary classification tasks, Gemini-Flash and GPT-4o both exhibited difficulties in fully capturing various sentence relations. For example, Gemini-Flash was able to identify the implication relation between NNA and Inference(a), but not the entailment relation when the sentence pair was reversed (Table 9). This inconsistency suggests that Gemini-Flash may have learned surface-level heuristics rather than deeper pragmatic representations, leading to a reliance on context-specific cues rather than a generalized understanding of entailment. Similarly, GPT-4o’s difficulties in consistently recognizing equivalence and its poor performance in distinguishing entailment relations when sentence pairs were swapped further highlight that these models lack a robust framework for understanding logical as well as pragmatically enriched relations between sentences.

We speculate several possible reasons for the LLMs’ shortcomings. First, LLMs may not be effectively learning the underlying semantic structures in language use, particularly where multiple interpretations are possible. The models’ reliance on context-specific patterns rather than generalizable reasoning steps could be a consequence of their training on large, diverse datasets that do not adequately emphasize formal semantic distinctions or pragmatic subtleties. Additionally, the lack of explicit training on tasks designed to capture these relationships may contribute to the observed variability and inconsistency in LLMs’ performance.

Overall, despite the careful control of input pairs and the specific instruction regarding the nature of entailment and implicature, LLM’s performance was sub-optimal, with accuracy rates below chance levels for both the baseline and target datasets. The lower accuracy and greater variation in responses to the baseline dataset suggest that LLM struggles with these nuanced semantic distinctions, particularly in the interpretation of manner implicatures. These reinforce the need for LLMs to incorporate more refined mechanisms that can handle complex inferential reasoning and context-dependent interpretations. To improve their capabilities, future LLMs development could benefit from integrating explicit semantic and pragmatic knowledge, as well as from training on datasets specifically curated to emphasize pragmatic reasoning. This would enable models to move beyond surface-level pattern recognition and towards a more robust understanding of language, which aligns more closely with human-like reasoning and formal linguistic theories.

## Limitations

One limitation of the current study lies in the scope of semantic relations examined. While the study focused on distinguishing between entailment, implied, and neutral relations, it did not explore a broader range of linguistic nuances, such as presuppositions or conversational implicatures beyond the target manner and baseline scalar implicatures. This narrow focus might have led to an incomplete understanding of how well LLMs capture the full spectrum of natural language meaning.

Additionally, the reliance on specific datasets for manner implicatures may not fully represent the diversity of language use, potentially limiting the generalizability of the findings. For example, SNLI may not be an ideal comparison baseline dataset, because it comes from a different corpus. As a consequence, the Spearman correlations seem very high, which might be indicating ceiling effects. This is also likely affected by sparse data. For next step research, we hope to have better control of the baseline dataset. Future research could benefit from incorporating a wider array of semantic phenomena and more diverse linguistic datasets to better assess the models’ capabilities.

Another limitation is the study’s reliance on standard evaluation metrics, such as cosine similarity and Spearman correlation, which may not fully capture the subtleties of semantic differentiation. These metrics, while useful, might oversimplify the complex interplay of meaning in language. Further, the observed instability in some models, particularly in newer LLMs such as Gemini-Flash, suggests that these models might have inherent variability that affects their performance in unpredictable and uninterpretable ways. This variability raises concerns about the reliability of the conclusions drawn from these models’ outputs. Addressing these issues might require the development of more refined evaluation techniques and a deeper investigation into the underlying causes of model instability, particularly in how LLMs encode and process pragmatic information.

## Conclusions

We proposed an approach to interacting with pre-trained LLMs from a linguistic perspective, particularly in pre-trained LLMs’ sensitivity to manner implicatures with different triggers. Some LLMs demonstrated some degree of sensitivity to some implicatures when using some of the metrics. We explored LLM explainability using an NLP pipeline capable of extracting pragmatically meaningful data points from large corpora. This pipeline enabled us to interpret LLMs’ outputs in a pragmatically sensible way. Grounded in formal and distributional linguistic observations, our experiments and results suggest that while there is potential in using pre-trained LLMs to characterize different shades of meaning, current models fall short in capturing the full complexity of language in use. Our findings indicate that pre-trained LLMs exhibit only minimal sensitivity to manner implicature. This suggests a need for the explicit inclusion of manner implicature datasets in their training, as well as careful refinement during pre-training. Overall, our results imply that the current paradigms in LLMs’ pre-training may be insufficient for fully capturing the diverse and subtle aspects of natural language meaning. We hope that our work can inspire future research on more effectively representing pragmatic reasoning in both theoretical and computational frameworks.

## Data Availability

The datasets consist of two types. The first type includes **target** datasets we created using specific templates. For manner implicatures triggered by causality and modality, we expanded a published experimental pragmatics stimulus set from Wilson^23^. For manner implicatures triggered by negation, we extracted sentences from the British National Corpus (BNC) written data^37^ that contain adjectives used in previous research by Aina^21^, which examined the distributional vector representations of 184 pairs of English adjectives. These **target** datasets are available online: https://osf.io/jp35t/. The second type, referred to as the **baseline** datasets, consists of existing, widely-cited datasets. This includes 1,199 entailment and implied sentence pairs extracted from a well-known scalar implicature dataset^4^: https://github.com/alexwarstadt/data_generation, focused on scalar implicatures triggered by quantifiers. Additionally, we included 1,094 sentence pairs with neutral relations, drawn from the Stanford Natural Language Inference corpus (SNLI)^42^: https://paperswithcode.com/dataset/snli. The script for the analysis in this paper is available online: https://osf.io/jp35t/.
